# Large Planar Na-β″-Al_2_O_3_ Solid Electrolytes for Next Generation Na-Batteries

**DOI:** 10.3390/ma13020433

**Published:** 2020-01-16

**Authors:** Samuel Clark Ligon, Marie-Claude Bay, Meike V. F. Heinz, Corsin Battaglia, Thomas Graule, Gurdial Blugan

**Affiliations:** 1 Laboratory for High Performance Ceramics, Empa, Swiss Federal Laboratories for Materials Science and Technology, Ueberlandstrasse 129, 8600 Duebendorf, Switzerland; thomas.graule@empa.ch (T.G.); gurdial.blugan@empa.ch (G.B.); 2 Materials for Energy Conversion, Empa, Swiss Federal Laboratories for Materials Science and Technology, Ueberlandstrasse 129, 8600 Duebendorf, Switzerland; marieclaude.bay@empa.ch (M.-C.B.); meike.heinz@empa.ch (M.V.F.H.); corsin.battaglia@empa.ch (C.B.)

**Keywords:** molten-salt batteries, sodium batteries, tape casting, planar cell, Na-β″-Al_2_O_3_

## Abstract

Large diameter (> 100 mm) planar Na-β″-Al_2_O_3_ solid electrolytes (BASE) with thickness from 1.0 to 1.5 mm have been prepared. Na-β″-Al_2_O_3_ was processed as a slurry and cast to give several meters of tape. One hundred and forty mm diameter discs were punched from the tape, stacked, and laminated with a large hydraulic press. Binder burnout and sintering were performed in 150 mm diameter MgO spinel encapsulations to mitigate the loss of Na_2_O vapor. Conductivity and flexural strength were measured on smaller Na-β″-Al_2_O_3_ samples produced via the same tape casting process followed by sintering and gave results consistent with BASE materials produced by uniaxial pressing of powders. Planar BASE membranes enable new cell designs, which are predicted to have higher power densities and better stacking efficiency compared to currently manufactured tubular cells.

## 1. Introduction

Sodium batteries have received increased interest in recent years principally for stationary energy storage, where load leveling is required to offset the intermittent nature of renewable energy sources (solar, wind, hydroelectric) and thus improve their implementation [1,2,3]. Na-β″-Al_2_O_3_ has high sodium-ion conductivity at elevated temperatures (0.20 S cm^−1^ at 300 °C) and is commercially utilized as the solid electrolyte in both sodium-sulfur (NaS) and sodium-nickel-chloride (NaNiCl) batteries [4,5,6]. Na-β″-Al_2_O_3_ is conventionally produced by calcining mixtures of Na_2_CO_3_ and Al_2_O_3_ powders [7]. The resultant alumina material should ideally have 8–9 wt% Na_2_O with a small portion (< 1.0%) of Li_2_O or MgO to stabilize the desired Na-β″-Al_2_O_3_ phase [8]. This powder is then further processed by milling and spray drying before pressing into the desired shape for the battery cell. A tubular design closed on one end is almost exclusively used for BASE membranes in commercial sodium batteries [1]. By comparison, planar BASE membranes have received rather limited attention even though they can potentially allow higher energy density cells and improve cell stacking efficiency [9].

One reason that planar BASE membranes have not received more attention is that commercially available planar membranes provide only a fraction of the surface area of commercial tubular and cloverleaf solid electrolytes. In prior work on the development of planar NaS and NaNiCl cells, Na-β″-Al_2_O_3_ membranes with a diameter of 45 mm were used [10,11]. This allows a maximum active surface area of less than 16 cm^2^, which is about 6% the active surface area of a commercial tubular cell (260 cm^2^). A theoretical planar circular cell with this same active area would require a membrane with a diameter of almost 200 mm. This is almost three times the maximum diameter (70 mm) currently offered by BASE manufacturers [12]. In a step towards the goal of producing planar cells with an active surface area comparable to commercial tubular cells, we describe herein the methodology to produce planar Na-β″-Al_2_O_3_ membranes with diameters of 110 mm. This provides a maximum active surface area of about 95 cm^2^, which is almost six times that of prior reported NaS planar cells and about a third the value of the commercial tubular cell. The proposed methodology is scalable and for the most part readily automated, which can help contribute the production of more affordable BASE membranes and ultimately more affordable sodium batteries.

## 2. Materials and Methods

### 2.1. Materials and Instrumentation

Li-stabilized Na-β″-alumina powder was prepared from boehmite (AlO(OH)), lithum hydroxide (LiOH), and sodium carbonate (Na_2_CO_3_) according to previously published methods [13,14]. More details on powder properties and sintered ceramics based on the same material can be found elsewhere [15]. All other chemicals were purchased from Sigma-Aldrich (Buchs, Switzerland). The laboratory table-top tape caster used was a model CAM-T0 from Keko Equipment (Zuzemberk, Slovenia). The large hydraulic press (5000 kN) was from Amsler-Laffon (Schaffhausen, Switzerland). Sintering was performed in a Nabertherm HT 40/17 furnace (Lilienthal, Germany) without purge gas.

Density was determined by the Archimedes method using a Mettler Toledo AG204 scale with MS-DNY-54 assembly (Greifensee, Switzerland). Flexural strength was measured by the ring-on-ring method using a Zwick Roell Z005 universal tester (Ulm, Germany) with a 5 kN Xforce P cell. The test specimens had a diameter of 25 mm, while the load and support diameters were 7 and 18 mm, respectively. A Netzsch STA449 F3 Jupiter instrument was used for differential scanning calorimetry/ thermal gravimetric analysis (DSC/TGA). Experiments were performed in static air with a heating rate of 5 °C min^−1^. X-ray diffraction was done with a Panalytical X’Pert Pro (Almelo, Netherlands) using CuKα radiation. The SEM used is a Tescan VEGA3 (Brno, Czechia) with a peripheral Bruker X Flash 6/0 EDS unit (Bruker Nano GmbH, Berlin, Germany). For cross sectional SEM images, the samples were first perpendicularly mounted in epoxy then ground and polished. Samples were sputtered immediately prior to SEM by 30 s exposure to a Pt/Pd alloy using a Cressington 108 Sputter Coater (Watford, UK).

### 2.2. Slurry Preparation and Tape Casting

The utilized slurry recipe was based on that of Lu et al. [16]. In a typical batch, 200 g of Na-β″-Al_2_O_3_, 27.0 g of poly(methyl methacrylate) (350 kDa), 12.2 g of di-butyl phthalate, and 12.7 g of PEG 300 were weighed and mixed in a 500 mL polypropylene container. To this was added an equal volume of 5 mm zirconia milling balls and a mixture of 51 g of ethanol and 104 g of 2-butanone (MEK). The mixture was ball milled with a speed of approximately 200 rpm for 24 h. Afterwards the slurry was filtered to remove the balls and rolled an additional 6 h to deaerate. The slurry was cast (Tape Caster Model CAM-T0 from Keko Equipment of Zuzemberk, Slovenia) using a 150 mm wide blade with a gap of 500 µm and a speed of 5 mm s^−1^. With these conditions, 240 g of slurry yielded approximately 2.5 m of tape. The tape was left to dry overnight at ambient conditions within the tape casting chamber.

### 2.3. Punching and Lamination

A 140 mm circular punch from LSB Stanzformen (Gunzgen, Switzerland) was used with a hand press to punch circular pieces of tape. To prepare a ceramic disc with thickness of 1.0–1.5 mm, 10 to 15 pieces were stacked within a stainless steel 142 mm diameter dye. The dye was then placed in an oven at 100 °C for 1 h and then pressed with 1.2 MN force to laminate the tape sections into a single green disc with a thickness from 1.6 to 2.0 mm depending on the number of layers used. Discs for mechanical testing were prepared in a similar fashion starting with a 32 mm diameter punch and dye. Rectangular samples (26 × 7 mm) for conductivity measurements were manually cut from the laminated green discs. For comparative purposes, samples were also prepared by uniaxial pressing of powder, in which case, discs for mechanical testing were formed by pressing approximately 3 g of powder with a 32 mm diameter dye using 100 MPa. Rectangular samples for conductivity measurements were pressed using approximately 0.90 g of powder with a dye (26 × 7 mm) and 180 MPa.

### 2.4. Debinding and Sintering

Prior to sintering, green Na-β″-Al_2_O_3_ discs were placed into 150 mm internal diameter MgO spinel crucibles (Morgan Haldenwanger, Waldkraiburg, Germany). Smaller spinel crucibles were used for sintering pieces for conductivity and mechanical analysis. The samples were placed on top of or covered with loose Na-β″-Al_2_O_3_ powder and first heated slowly to burn out organics (see Table 1 for further information). A heating rate of 0.5 °C min^−1^ was used up to 500 °C with a 2.5 h dwell at 200 °C. Above 500 °C, the rate was increased to 3 °C min^−1^ with a 2 h dwell at 1000 °C. Sintering was performed for 5 min at 1600 °C. Cooling to ambient temperature was performed with a rate of 3 °C min^−1^. Afterwards, extraneous Na-β″-Al_2_O_3_ powder was removed from the surface of sintered discs by sanding with SiC paper.

### 2.5. Na^+^ Conductivity Measurement

Temperature-dependent ionic conductivity was measured by electrochemical impedance spectroscopy with a Zahner IM6ex instrument (Kronach, Germany). A four-point-probe configuration with four isolated Pt wires linearly aligned with fixed separations (5; 6; 5 mm) was used. Prior to measurement, a colloidal graphite suspension (Aquadag) mixed with NaNO_3_ and NaNO_2_ was applied to the rectangular samples at the points of contact. The samples were heated within a tube furnace (Carbolite HST/200 from Carbolite Gero Ltd. of Hope Valley, UK) to 350 °C before slowly cooling to room temperature. Impedance was measured every 5 min from 1 Hz to 1 MHz with an amplitude of 20 mV. Temperature was tracked with a data logger (EL-USB-TC from Lascar Electronics of Essex, UK) and a digital thermometer (TM-947SD from Lutron of Coopersburg, PA, USA). The corresponding impedance features constant values at phase angles close to 0° for frequencies below 10 kHz, which is ascribed to the total resistance R of the electrolyte. From the measured resistance at 10 kHz, the sample conductivity σ = (1/R)·(L/A) was calculated using the cross-sectional area of the sample (A) and the distance between the two inner electrodes (L).

### 2.6. Sample Dimensions and Tolerance Determination

Disc diameter was measured with calipers at angles of 0, 45, 90, and 135°. Thickness was measured with a digital micrometer (IP 65 from Mitutoyo, Urdorf, Switzerland). In both cases, the average of at least four measurements is expressed with half the difference between the minimum measured value and the maximum measured value provided to define the tolerance for thickness. Plane parallelity (PP) was determined with a Mitutuyo LH-600 linear height gauge (Neuss, Germany). For each sample, the linear displacement from the top surface to the flat reference surface was measured at ten points. Plane parallelity is expressed as the difference between the maximum measured displacement and the average measured thickness.

## 3. Results and Discussion

### 3.1. Tape Casting and Warm Lamination

**Figure 1:**
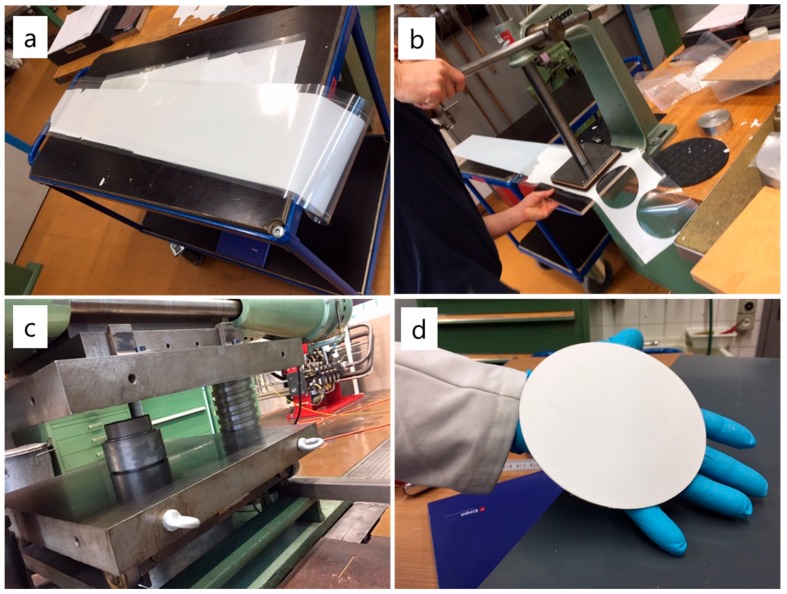
Production of large diameter Na-β″-Al_2_O_3_ discs. (**a**) Several meters of tape collected; (**b**) punching 140 mm discs; (**c**) warm pressing with 1.2 MN; (**d**) 1.9 mm thick green disc ready to be sintered.

Commercial tubular BASE membranes are generally produced by isostatic dry bag pressing, although Ionotec also uses electrodeposition and extrusion for the production of certain geometries [17]. By comparison, tape casting is a commercially established method for producing flat ceramic sheets for applications including microelectronic circuits and fuel cells [18,19,20]. Lu et al. describe tape casting of Na-β″-Al_2_O_3_, where they found that PMMA binder with a mixed MEK/ethanol solvent system provided much more consistent results than tapes based on PVB in water [16]. We used a similar slurry recipe substituting PEG 300 for PEG 600 and increasing the ratio of DBP to improve plasticity. The slurry was roll milled for 24 h to break up any aggregates and homogenize before filtering and rolling again for a few hours to deaerate. Tape casting was performed with a casting blade slightly wider (150 mm) than the target diameter. The tape was collected atop a 200 mm wide polyethylene film with a silicone coating to reduce adhesion. An example of a tape is presented in Figure 1a. Overnight ambient drying was found to be sufficient to give crack-free tapes that were easy to remove from the film and sufficiently flexible for punching and lamination. The thickness of the resultant tape was approximately 250 µm.

Two to three meters of tape were spooled to simplify transportation between the tape casting instrument and the hand press. Circular pieces of tape were cut with a 140 mm punching tool (Figure 1b). The circular tapes tended to release on their own from the polyethylene carrier film. The pieces were stacked within a 142 mm stainless steel dye using polyethylene pieces on the top and bottom to prevent adhesion. The dye was assembled and placed in an oven at 100 °C for 1 h before laminating with a large hydraulic press (Figure 1c). A force of 1.2 MN was used, which gave green discs from 1.5 to 2.0 mm thickness depending on the number of tape pieces being laminated (Figure 1d).

### 3.2. Debinding and Sintering

**Figure 2:**
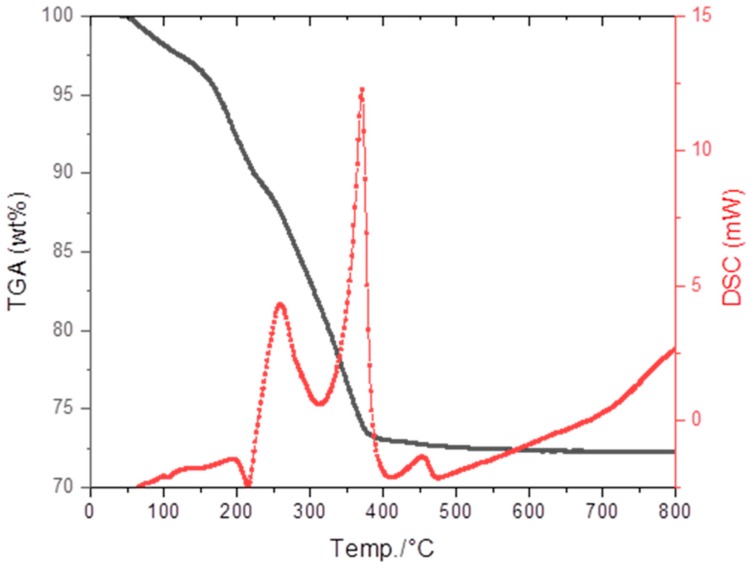
Differential scanning calorimetry/ thermal gravimetric analysis (TGA/DSC) of Na-β″-Al_2_O_3_ tape. The black curve and left axis display thermal gravimetric weight loss, while the red curve and right axis display the DSC data (exotherm up).

Residual tape was used for thermal analysis (DSC/TGA) to determine optimal conditions for binder removal and sintering. As Figure 2 shows, the tape contains almost 30 wt% organics. To avoid cracks and warpage, the binder must be burned out very slowly. Thus, at temperatures below 500 °C, a heating rate of 0.5 °C min^−1^ was used with a 2.5 h dwell at 200 °C. Above 500 °C, no weight loss is observed and heating could be performed rather quickly (3 °C min^−1^). We included an additional dwell of 2 h at 1000 °C to presinter the pieces before sintering at 1600 °C. A dwell of only 5 min was used, since longer times have in previous studies found to contribute to rapid and inhomogeneous grain growth, which tends to negatively affect mechanical properties [21].

**Figure 3:**
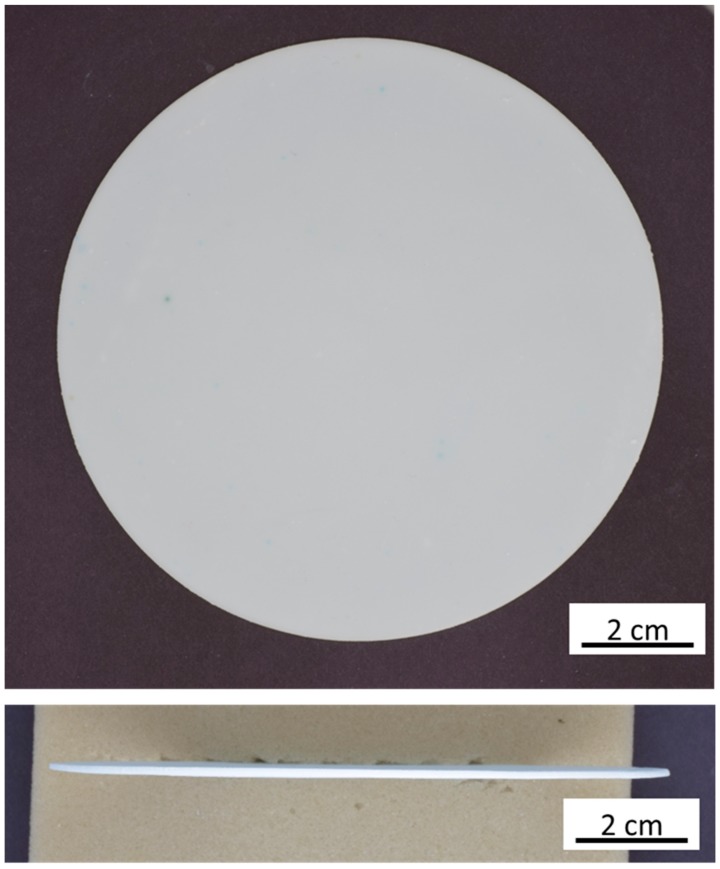
One hundred and ten mm diameter planar BASE membrane after sintering.

Sintering was performed within custom-made 150 mm internal diameter MgO spinel crucibles. To prevent evaporation of Na_2_O during sintering, the discs were buried with an excess of loose Na-β″-Al_2_O_3_ powder and enclosed with spinel cover plates. Some of the powder will inevitably fuse to the disc during sintering, but it was found to be easily removed by polishing with SiC paper. The resultant planar membrane is flat and crack-free (Figure 3). The diameter and thickness of discs before and after sintering were measured to calculate linear shrinkage in both x/y- and z-directions (Table 1). In spite of the relatively high concentration of organic binder used, sintering-associated shrinkage was fairly comparable for Na-β″-Al_2_O_3_ ceramics prepared via both pathways. Shrinkage was fairly isotropic for the samples prepared by pressing. By comparison, linear shrinkage was significantly greater in the z-direction compared to x and y for the discs prepared through tape casting and lamination.

Large diameter BASE membranes have been produced for use in planar sodium batteries. For use in a functional cell, it is important to accurately measure all dimensions and to define tolerance zones so that the discs can fit within the device. Table 1 provides dimensions with errors for the first six discs produced. Differences in diameter between entries are due to slight differences in tape composition. Importantly, in all cases the discs have good roundness with tolerance values between 0.1% and 0.2% for the diameter. Disc thickness is dependent on tape casting conditions and most importantly on the number of layers laminated. We chose to produce discs with different thicknesses to test the flexibility of the process, since the optimal thickness for the device has yet to be determined. More importantly, the measured tolerances for thickness were in all cases low. While the discs were round and uniform in thickness, puckering (expressed quantitatively as a high plane parallelism value) was a problem in early batches. This problem was partially resolved by sintering the discs directly on the surface of the spinel crucible. Covering the disc with presintered as opposed to fresh β″-Al_2_O_3_ powder was also found to have a positive influence on disc flatness.

### 3.3. Mechanical and Electrical Properties

Rather than attempting to measure the mechanical properties of the large diameter planar BASE membranes, smaller discs were prepared through the same methodology. In which case, a smaller punch and dye were used to make green laminated discs with 32 mm diameters. The sintered test discs had a typical diameter of 25 mm and thickness of 1.4 mm. For comparison purposes, multiple discs with the same dimensions were prepared through a traditional powder pressing process. Sintering conditions (1600 °C for 5 min) were the same for all of the discs. Ring-on-ring flexural strength testing revealed that the Na-β″-Al_2_O_3_ discs prepared by tape casting and lamination were slightly stronger than those prepared by powder pressing (144 MPa vs. 127 MPa). The density of the samples was determined from the broken pieces using the Archimedes method in water. The relative density of the discs prepared by powder pressing and those prepared by tape casting were found to be comparable (Table 2).

**Figure 4:**
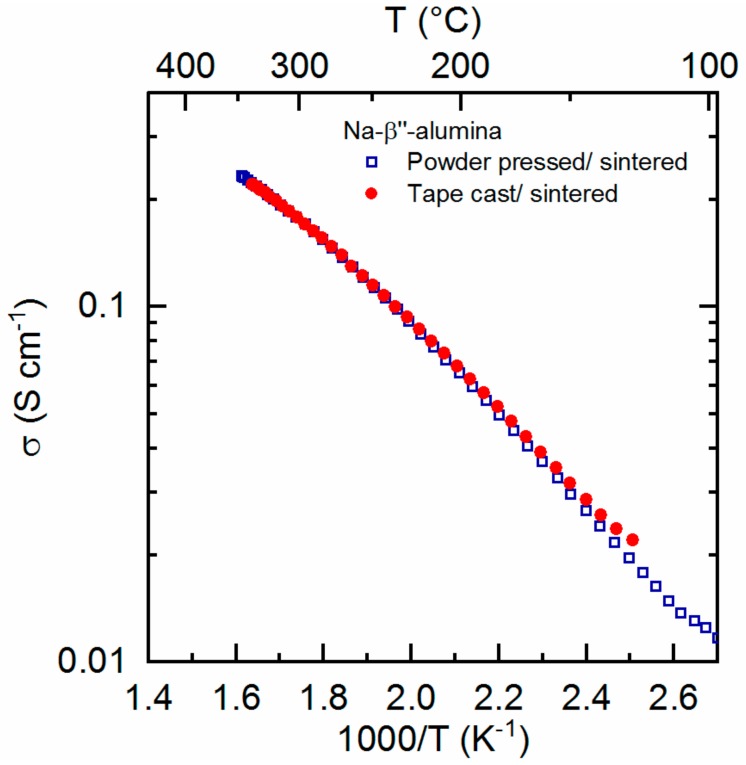
Na+ conductivity of Na-β″-Al_2_O_3_ ceramics prepared by uniaxial powder pressing and sintering (blue squares) vs. Na-β″-Al_2_O_3_ ceramics prepared by tape casting and sintering (red dots).

Similar to the preparation of samples for mechanical testing, rectangular pieces were cut from green laminated discs and sintered to give bars for four-point conductivity measurements. Conductivity was measured on cooling from 350 °C and found to match the data of a Na-β″-Al_2_O_3_ bar prepared by a traditional powder pressing/sintering pathway (Figure 4). Conductivity of both samples was 0.18 S cm^−1^ at 300 °C, which is a standard operating temperature for a sodium battery. The apparent activation energies (*E_a_*) for both samples were determined by linear interpolation of the conductivity data.

(1) $$\sigma T=\;\sigma_{0}\exp\left(-\frac{E_{a}}{k_{b}T}\right)$$

Here, *σ*_0_ is a pre-exponential factor and *k_b_* is Boltzmann’s constant. An activation energy of 0.29 eV was calculated for both samples. This correlates quite well with values given by Virkar et al. when comparing fine grained lithium stabilized Na-β″-Al_2_O_3_ (0.221 eV) and polycrystalline samples (0.332 eV) [13].

### 3.4. Morphology and Microstructure

**Figure 5:**
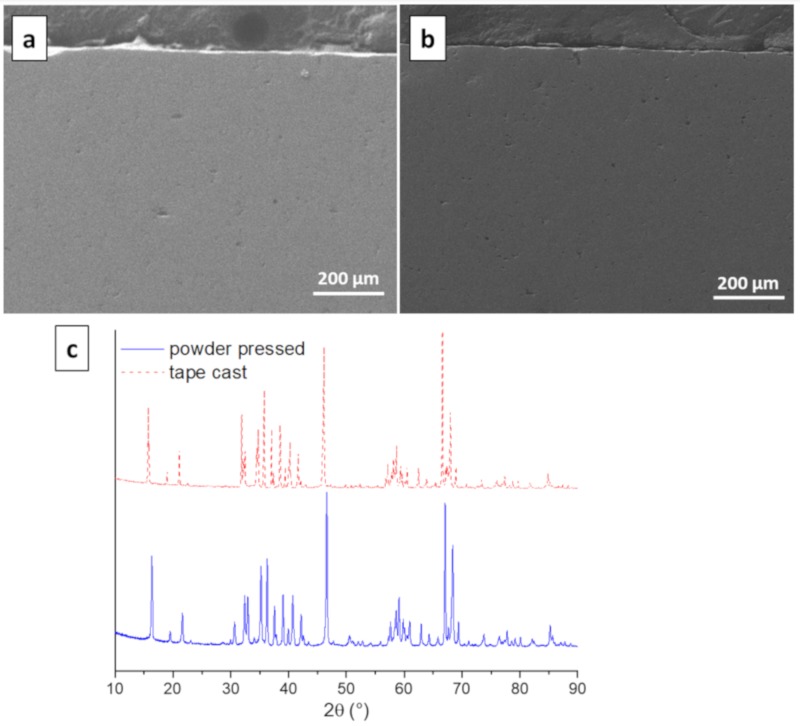
SEM of Na-β″-Al_2_O_3_ ceramics prepared by uniaxial powder pressing and sintering (**a**) and by tape casting and sintering (**b**). XRD of both (**c**).

SEM and XRD were performed on Na-β″-A_l2_O_3_ ceramics prepared by both conventional powder pressing and by tape casting. For SEM, the samples were mounted perpendicularly in epoxy resin and polished to view the cross sections. This was done principally to ensure that no horizontal striations remained after sintering the laminated green pieces. As seen in Figure 5a,b, both samples are homogenous and look essentially the same. Moreover, at higher magnifications (5000×), no differences were noted. The XRD patterns of the two Na-β″-Al_2_O_3_ ceramics also look very similar, although the powder pressed sample exhibits small peaks at 30 and 50° (Figure 5c). These peaks are indicative of β-Al_2_O_3_, which can form from Na-β″-Al_2_O_3_ during sintering without excess Na_2_O [21]. The peaks were not seen for the samples prepared via tape casting and lamination, since the green pieces were buried in excess Na-β″-Al_2_O_3_ during sintering.

## 4. Conclusions

A multistep process consisting of tape casting, punching, lamination, and sintering has been used to prepare large (> 100 mm) diameter BASE membranes for planar sodium battery cells. Sintering conditions were optimized with the help of DSC and TGA to provide flat and crack-free ceramics. Test pieces fabricated using the same methodology were used to compare with Na-β″-Al_2_O_3_ samples prepared by a conventional powder pressing pathway. The ceramics prepared through the tape casting pathway were slightly denser and stronger and had conductivities virtually identical to the control samples. The processing technologies presented (tape casting, punching, lamination) are scalable and can be readily automated, which should help in the production of larger BASE membranes at lower costs. This work should help enable planar sodium cells with active surface areas approaching that of commercial tubular cells. The development of a functional planar cell based on membranes with diameters greater than 100 mm is currently underway.

## Figures and Tables

**Table 1:** Effect of sintering conditions on Na-β″-Al_2_O_3_ ceramic disc dimensions. Diameter is expressed as the average of four measurements made at 0, 45, 90, and 135°. The error value is half of the difference between the maximum and minimum measured value and provides a roundness tolerance. Thickness is expressed as the average of five measurements made with a digital micrometer. The error value is half of the difference between the maximum and minimum measured value. Plane parallelism (PP) is expressed as the difference between the sample thickness and the maximum measured displacement from the top surface of the sample to the flat reference surface beneath.

Sintering Conditions	Diameter (mm)	Thickness (mm)	PP (mm)
Atop fresh β″-Al_2_O_3_ powder	109.15 ± 0.15	1.61 ± 0.079	0.93
Atop fresh β″-Al_2_O_3_ powder	109.17 ± 0.23	1.41 ± 0.098	1.6
Atop presintered β″-Al_2_O_3_ powder	110.44 ± 0.16	1.63 ± 0.098	1.8
Atop fresh/presintered β″-Al_2_O_3_ mix	111.29 ± 0.19	1.38 ± 0.038	1.0
Beneath fresh β″-Al_2_O_3_ powder	110.04 ± 0.08	1.59 ± 0.055	1.0
Beneath presintered β″-Al_2_O_3_ powder	109.99 ± 0.15	1.16 ± 0.023	0.13

**Table 2:** Comparison of properties of Na-β″Al_2_O_3_ ceramics formed by uniaxial pressing of powder versus those prepared by tape casting and lamination. Sintering conditions were 1600 °C for 5 min in all cases.

	Powder Pressing	Tape Casting/Lamination
Sinter shrinkage (x/y)	23.2%	22.9%
Sinter shrinkage (z)	23.1%	27.3%
Relative density (%)	96.8%	98.1%
Flexural strength (MPa)	127 ± 13	144 ± 14
Conductivity at 300 °C (S cm^−1^)	0.18	0.18
